# Proposal of Furfurilactobacillus cerevisiae sp. nov. isolated from spoiled beer and Furfurilactobacillus cerealis sp. nov. isolated from sourdough

**DOI:** 10.1099/ijsem.0.007009

**Published:** 2025-12-18

**Authors:** Dor Zipori, Nanzhen Qiao, Merlin Brychcy, Michael G. Gänzle, Herbert Schmidt

**Affiliations:** 1 Dept. of Food Microbiology and Hygiene, Institute of Food Science and Biotechnology, University of Hohenheim, Stuttgart, Germany; 2 Dept. of Agricultural, Food and Nutritional Science, University of Alberta, Edmonton, AB, Canada

**Keywords:** average nucleotide identity (ANI), digital DNA–DNA hybridization (dDDH), *Furfurilactobacillus cerealis*, *Furfurilactobacillus cerevisiae*, novel species

## Abstract

A core genome phylogenomic analysis of two lactic acid bacteria isolates from spoiled beer, *Furfurilactobacillus* sp. LTH 5742 and LTH 5750, one isolate from sourdough, *Furfurilactobacillus* sp. C5, and the type strains of five *Furfurilactobacillus* species were performed. Average nucleotide identity and digital DNA–DNA hybridization analyses revealed two distinct phylogenetic lineages, both clearly separated from the five previously described *Furfurilactobacillus* species. Based on these data, two novel species were proposed: *Furfurilactobacillus cerevisiae* sp. nov., represented by strains LTH 5742^T^ (=DSM 120514^T^=LMG 34151^T^) and LTH 5750, and *Furfurilactobacillus cerealis* sp. nov., represented by strain C5^T^ (=DSM 120515^T^=LMG 34152^T^). Comparative genomic analyses highlighted distinct metabolic signatures concerning the 1,2-propanediol and hydroxycinnamic acid metabolism. The gene *aldA* encoding lactaldehyde dehydrogenase, contributing to 1,2-propanediol production from lactate, is present in *Ff*. spp. LTH 5742^T^ and LTH 5750, *Ff*. spp. C5^T^ and *Furfurilactobacillus rossiae* but not in *Furfurilactobacillus milii*. Genes for 1,2-propanediol conversion to propionate and propanol are present in *Ff. rossiae* but not in one of the other species. The glutathione reductase gene, *gshR*, was uniquely detected in *Ff. cerealis*, *Ff. rossiae* and ‘*Furfurilactobacillus entadae’*. These findings expand the diversity of the genus *Furfurilactobacillus* and provide insight into their ecological adaptation in cereal-based fermentations.

## Introduction

The genus *Furfurilactobacillus* was formally established in 2020 following the taxonomic reclassification of the former *Lactobacillus rossiae* group [1]. At its introduction, it comprised three heterofermentative species previously classified as *Lactobacillus: Lactobacillus rossiae* [2], *Lactobacillus siliginis* [3] and *Lactobacillus curtus* [4]. The genus has since been expanded with the description of *Furfurilactobacillus milii* in 2022 [5] and the proposed species ‘*Furfurilactobacillus entadae*’ in 2025 [6].

Members of this genus have been isolated from a broad range of environments, including plant materials, sourdough and other cereal fermentations, beer, fermented meat products and animal faeces [1,5, 6].

Many *Furfurilactobacillus* species are capable of degrading phenolic compounds, reflecting their ecological adaptation to plant-derived substrates [7]. In addition, several strains also exhibit traits of technological importance in food fermentation, such as antifungal activity and vitamin biosynthesis [8,9]. Conversely, some members also contribute to beer spoilage. For example, certain *Furfurilactobacillus curtus* and *Furfurilactobacillus rossiae* strains persist and proliferate under selective conditions that typically inhibit bacterial growth, including the presence of ethanol and hop-derived antimicrobial compounds [4,10]. Moreover, some *Furfurilactobacillus* spp. strains produce exopolysaccharides (EPSs), which can negatively affect beer quality by increasing viscosity and turbidity, though EPS production can be advantageous in other fermented foods by enhancing texture and product stability [10,11].

When introduced in 2022, *Ff. milii* comprised a diverse and physiologically heterogeneous group of strains associated with cereal fermentations, including strain C5 [5]. Distinction of *Ff. milii* from *Ff. rossiae* could not be resolved using 16S rRNA gene sequencing alone, which made whole-genome analysis necessary to establish the species. More recently, two strains LTH 5742 and LTH 5750 were reported to produce EPSs and were initially assigned to the species *Ff. rossiae* based on 16S rRNA gene similarity [11].

In the present work, we describe the genomic and physiological characterization of the *Furfurilactobacillus* strains C5^T^, LTH 5742^T^ and LTH 5750 and propose two novel species: *Furfurilactobacillus cerevisiae* sp. nov. and *Furfurilactobacillus cerealis* sp. nov.

## Isolation and ecology

The strains LTH 5742^T^ and LTH 5750 were isolated in 2002 from two different spoiled beer samples, originating from different batches of the same brewery [11]. Both appeared as white, slimy colonies on de Man–Rogosa–Sharpe (MRS) agar. The strain C5 was isolated from Italian sourdough in 2012 on modified MRS agar [7]. Both isolation sources reflect ecological niches typical for the genus *Furfurilactobacillus*, spanning both cereal fermentations and beer environments [1].

## Genomic analyses

**Fig. 1.**
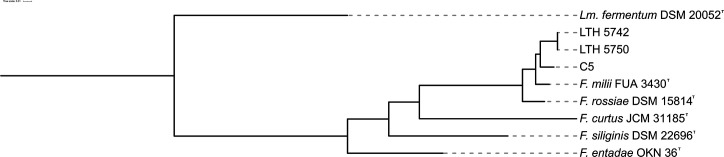
Core genome approximate maximum-likelihood phylogenetic tree of LTH 5742^T^, LTH 5750, C5^T^, *Ff. milii* FUA 3430^T^, *Ff. rossiae* DSM 15814^T^, *Ff. curtus* JCM 31185^T^, *Ff. siliginis* DSM 22696^T^, *‘Ff. entadae’* OKN 36^T^ and the outgroup *Lm. fermentum* DSM 20052^T^, based on 396 core genes, with a 60% identity threshold in Roary. The phylogenetic tree was visualized using iTOL [15]. Bootstrap support for all nodes was greater than 95%.

Bacterial strains and corresponding genome sequences used in this study are listed in Table 1. Genomes were annotated using Prokka v1.14.6 [12]. Core-genome alignment was generated using Roary v3.11.2 [13] with the identity threshold of 60%. With *Limosilactobacillus fermentum* DSM 20052^T^ rooted as the outgroup, a set of 396 core genes was identified and used to infer the maximum-likelihood phylogenies with FastTree v2.2.0 under the JTT model [14]. The final phylogenetic tree was visualized on the Interactive Tree of Life (iTOL) platform [15] (Fig. 1). Strains LTH 5742^T^ and LTH 5750 formed a monophyletic cluster, distinct from all currently described *Furfurilactobacillus* species (Fig. 1). Their closest relative was strain C5^T^, which itself branched separately from *Ff. milii* FUA 3430^T^. The three strains LTH 5742^T^, LTH 5750 and C5^T^ formed a phylogenetic clade with *Ff. milii* and *Ff. rossiae* which was separated from the type strains of *Ff. curtus*, *Furfurilactobacillus siliginis* and ‘*Ff. entadae’* (Fig. 1).

**Table 1.** Strains and genome sequences used in this study

Strain	Assembly accession	Species	Isolation source	Ref
DSM 20052^T^	GCA_013394085.1	*Lm. fermentum*	–	
LTH 5742^T^	GCA_048961765.1	*Ff. cerevisiae*	Spoiled beer	[11]
LTH 5750	GCA_049064545.1	*Ff. cerevisiae*	Spoiled beer	[11]
C5^T^	GCA_009864005.1	*Ff. cerealis*	Sourdough	[5]
FUA 3430^T^	GCA_021654115.1	*Ff. milii*	Sourdough	[5]
FUA 3115	GCA_021646505.1	*Ff. milii*	Sourdough	[5]
FUA 3119	GCA_021646525.1	*Ff. milii*	Sourdough	[5]
DSM 15814^T^	GCA_001435135.1	*Ff. rossiae*	Sourdough	[8]
JCM 31185^T^	GCA_045862785.1	*Ff. curtus*	Beer	[4]
DSM 22696^T^	GCA_001437435.1	*Ff. siliginis*	Sourdough	[28]
OKN 36^T^	GCA_045862745.1	*‘Ff. entadae’*	*Entada phaseoloides*	[6]

Analyses of 16S rRNA gene identity and 16S rRNA gene-based phylogeny do not resolve the relationships among *Ff. rossiae* and closely related species [5,11]. To determine the taxonomic status of the three strains of *Furfurilactobacillus* spp., the pairwise average nucleotide identities (ANIs) were calculated using OrthoANI [16]. Digital DNA–DNA hybridization (dDDH) values were estimated using the DSMZ’s Genome-to-Genome Distance Calculator v3.0 [17]. The strains LTH 5742^T^ and LTH 5750 are highly similar, with strain C5^T^ representing the most closely related genome/novel species, followed by *Ff. milii* FUA 3430^T^ (Fig. 1, Tables2  3). The ANI values between strain C5^T^ to *Ff. milii* FUA 3430^T^, as well as between strains LTH 5742^T^ and LTH 5750 and C5^T^, were ~94.5%, which is below the established species delineation threshold of 95–96% [18]. dDDH analysis further supported this distinction. The strains LTH 5742^T^ and LTH 5750 shared ~58% dDDH with C5^T^ and ~55% with *Ff. milii* FUA 3430^T^, while C5^T^ shared a 60.1% dDDH with *Ff. milii* FUA 3430^T^. These values are well below the 70% threshold that has served as the criterion for species delineation [18].

**Table 2.** ANI percentages between *Furfurilactobacillus* spp. LTH 5742^T^, LTH 5750, C5 and type strains of other five *Furfurilactobacillus* species

	LTH 5742^T^	LTH 5750	C5^T^	FUA3430^T^	DSM 15814^T^	JCM 31185^T^	DSM 22696^T^	OKN 36^T^
LTH 5742^T^	–							
LTH 5750	99.97	–						
C5^T^	94.60	94.50	–					
*Ff. milii* FUA3430^T^	93.77	93.78	94.96	–				
*Ff. rossiae* DSM 15814^T^	92.50	92.61	92.65	92.63	–			
*Ff. curtus* JCM 31185^T^	72.94	73.26	72.60	72.63	72.64	–		
*Ff. siliginis* DSM 22696^T^	74.66	74.71	74.55	74.09	74.12	72.71	–	
*‘Ff. entadae’* OKN 36^T^	73.50	73.60	73.49	73.02	73.21	71.83	73.85	–

**Table 3.** dDDH identities between *Furfurilactobacillus* spp. LTH 5742^T^, LTH 5750, C5^T^ and type strains of other *Furfurilactobacillus* species

	LTH 5742^T^	LTH 5750	C5^T^	FUA3430^T^	DSM 15814^T^	JCM 31185^T^	DSM 22696^T^	OKN 36^T^
LTH 5742^T^	–							
LTH 5750	100.00	–						
C5^T^	57.80	57.90	–					
*Ff. milii* FUA3430^T^	54.70	55.00	60.10	–				
*Ff. rossiae* DSM 15814^T^	49.20	49.40	48.90	49.00	–			
*Ff. curtus* JCM 31185^T^	29.90	29.60	22.20	22.00	21.10	–		
*Ff. siliginis* DSM 22696^T^	27.60	27.50	22.00	22.20	20.90	22.80	–	
*‘Ff. entadae’* OKN 36^T^	20.50	20.50	19.80	20.10	20.20	19.70	20.60	–

Genomic features were consistent with these phylogenomic relationships inferred from ANI and dDDH. Within the lineage comprising the two LTH strains, C5^T^, *Ff. milii* and *Ff. rossiae*, LTH 5742^T^ and LTH 5750 possessed the largest genome, averaging 2.9 Mbp, followed by *Ff. rossiae* (2.9 Mbp) and strain C5^T^ (2.7 Mbp). *Ff. milii* had the smallest genome in this clade (2.6 Mbp) (Table 4). The genomes of the more distantly related species *Ff. curtus* JCM 31185^T^, *Ff. siliginis* DSM 22696^T^ and ‘*Ff. entadae’* OKN 36^T^ were smaller, ranging from 2.1 to 2.2 Mbp. The G+C content of LTH 5742^T^, LTH 5750 and C5^T^ was ~43.5mol%, identical to the values of closely related type strains of *Ff. milii* and *Ff. rossiae*, but slightly lower than the values observed for *Ff. curtus* (44%), *Ff. siliginis* (44mol%) and ‘*Ff. entadae’* (47mol%) (Table 4).

**Table 4.** Genomic features of *Furfurilactobacillus* spp. LTH 5742^T^, LTH 5750, C5^T^ and type strains of other *Furfurilactobacillus* species

Strain	Assembly accession	Species	Genome size (Mbp)	G+C content (mol%)
LTH 5742^T^	GCA_048961765.1	*Ff. cerevisiae*	3.00	43.5
LTH 5750	GCA_049064545.1	*Ff. cerevisiae*	2.91	43.5
C5^T^	GCA_009864005.1	*Ff. cerealis*	2.73	43.5
FUA 3430^T^	GCA_021654115.1	*Ff. milii*	2.65	43.5
DSM 15814^T^	GCA_001435135.1	*Ff. rossiae*	2.87	43.5
JCM 31185^T^	GCA_045862785.1	*Ff. curtus*	2.18	44.0
DSM 22696^T^	GCA_001437435.1	*Ff. siliginis*	2.07	44.0
OKN 36^T^	GCA_045862745.1	*‘Ff. entadae’*	2.26	47.0

To identify genomic traits distinguishing the proposed novel species from closely related species, gene presence/absence analyses were conducted. GFF files generated by Prokka [12] were used as input for Roary [13] to generate the pan-genome, and species-level patterns were explored using Scoary v1.6.16 [19] comparing two LTH strains with all remaining strains. Genes uniquely associated with the LTH lineage as indicated by Scoary, together with other key enzymes typically implicated in carbohydrate metabolism, stress tolerance and adaptation to plant-associated environments [5,7, 20,23], were further investigated by blastp [24] searches with thresholds of ≥40% identity and ≥70% coverage (Table 5).

**Table 5.** Distribution of genes coding for metabolic enzymes in *Furfurilactobacillus* spp. LTH 5742^T^, LTH 5750, C5^T^ and type strains of other *Furfurilactobacillus* species Grey shading and numbers represent the presence and gene copy numbers of the genes.

		*Ff. cerevisiae*	*Ff. cerevisiae*	*Ff. cerealis*	*Ff. milii*	*Ff. rossiae*	*Ff. curtus*	*Ff. siliginis*	*‘Ff. entadae’*
		LTH 5742^T^	LTH 5750	C5^T^	FUA 3430^T^	DSM 15814^T^	JCM 31185^T^	DSM 22696^T^	OKN 36^T^
Lactaldehyde dehydrogenase *aldA*	KRM30456.1	1	1	1	0	1	0	0	0
Diol dehydratase, *pduCDE*	CAC82541.1, CAC82542.1, CAD01091.1	0	0	0	0	1	0	1	1
*β*-Glucosidase *bglA*	WP_178941777.1	1	1	1	1	1	1	1	0
*β*-Glucosidase *bglB*	WP_017261864.1	1	1	1	1	1	1	1	0
*β*-Glucosidase *bglC*	WP_178943320.1	1	1	1	1	1	1	1	0
*β*-Glucosidase *bglD*	WP_178942543.1	1	1	1	1	1	1	1	0
Glutathione reductase, *gshR*	ABI79324.1	0	0	1	0	1	0	0	1
Hydroxycinnamic acid decarboxylase *pad*	WP_003641609.1	1	1	0	1	1	0	0	1
Hydroxycinnamic acid reductase *par1*	WP_161000921.1	1	1	1	1	1	1	1	0
Hydroxybenzoic acid decarboxylase *lpdC*	WP_003644796.1	1	1	1	0	0	0	0	1
Vinyl phenol reductase *vprA*	YP_004888771.1	0	0	0	1	1	0	0	1

The lactaldehyde dehydrogenase encoding gene *aldA* was identified in the two LTH strains, C5^T^ and *Ff. rossiae*, but not in *Ff. milii* FUA3430^T^. Previous reports that described its presence in both *Ff. milii* and *Ff. rossiae* assigned *Ff. cerealis* C5^T^ to *Ff. milii* [5]. The proposal of *Ff. milii* sp. nov. also included *Ff. milii* FUA 3115 and FUA 3119. To re-evaluate this discrepancy, multiple lactaldehyde dehydrogenase query sequences from *Furfurilactobacillus* species or from different genera (accessions: MYV05750.1, MYV17209.1, J9W2N6, KRM30456.1, CCK23494.1 and CAJ1229574.1) were used for blastp validation. The gene *aldA* encoding lactaldehyde dehydrogenase was absent in *Ff. milii* FUA 3430^T^, FUA 3115 and FUA 3119 (Table 5 and data not shown). The diol utilization operon, including the diol dehydratase *pduCDE,* was absent in *Ff. cerevisiae* LTH 5742^T^ and LTH 5750, *Ff. cerealis* C5^T^, *Ff. milii* and *Ff. curtus*, but present in *Ff. rossiae*, *Ff. siliginis* and ‘*Ff. entadae’*. The presence of both lactaldehyde dehydrogenase and diol dehydratase enables conversion of lactate to acetate, propionate and propanol with 1,2-propanediol as metabolic intermediate [21,25]. To date, conversion of lactate to propionic acid in a single organism was reported only for *Levilactobacillus lettrarii* [25].

To determine whether the conversion of lactate to 1,2-propanediol and the conversion of diols by diol dehydratase are phylogenetic markers which inform on the taxonomic position of the genus *Furfurilactobacillus*, we analysed all 34 genomes of *Furfurilactobacillus* spp. which were available in November 2025 (Fig. S1, available in the online Supplementary Material). This analysis revealed that lactate and diol catabolism is a trait that is shared among all strains of a species. *Ff. rossiae* is the only species that converts lactate to propionate. *Ff. cerealis* and *Ff. cerevisiae* and an undescribed species convert lactate to 1,2-propanediol. *Ff. siliginis* and *‘Ff. entadae*’ convert 1,2-propanediol to propionate. With the exception of *Ff. rossiae*, where the conversion of diols has been shown experimentally [26], documentation that the phenotype matches the genotype remains subject to future studies.

Genes encoding *β*-glucosidases [23] were present in *Ff. cerevisiae* LTH 5742^T^ and LTH 5750, *Ff. cerealis* C5^T^, *Ff. milii*, *Ff. rossiae*, *Ff. curtus* and *Ff. siliginis* but not in ‘*Ff. entadae’* (Table 5). These enzymes, associated with the hydrolysis of plant glycosides, indicate adaptation of the clade including *Ff. cerevisiae*, *Ff. cerealis, Ff. milii* and *Ff. rossiae* and plant-associated niches that offer glycosylated phytochemicals as substrates [23]. A glutathione reductase homologue was identified exclusively in *Ff. cerealis* C5^T^, *Ff. rossiae* and ‘*Ff. entadae’*. Of the genes involved in the metabolism of hydroxycinnamic acids, the hydroxycinnamic acid decarboxylate *pad*, the vinyl phenol reductase *vprA*, the hydroxycinnamic acid reductase *par1* and the hydroxybenzoic acid decarboxylate *lpdC* were detected and were differentially present in type strains of *Furfurilactobacillus* species (Table 5).

## Morphology and physiology

The strains LTH 5742^T^, LTH 5750 and C5^T^ were cultivated in MRS medium (Merck KGaA) and incubated at 30 °C under anaerobic conditions in sealed jars using Anaerocult® A system (Merck KGaA). Colonies of *Ff. cerevisiae* strains grown on MRS agar for 72 h were 0.5–1.0 mm in diameter, white, smooth, circular and convex with a shiny and sticky surface. The ropy or slimy colony phenotype is a strain-specific characteristic associated with the production of cell wall-associated EPSs. In this case, the phenotype has been linked to a plasmid-encoded glycosyltransferase [11]. While it is stably maintained on agar plates, the ropy phenotype can disappear after repeated subculturing in liquid medium. This behaviour is consistent with observations reported for other ropy lactobacilli [27]. Examination of *Ff. cerevisiae* LTH 5742^T^ under light microscope showed Gram-positive cells, ~1.5 µm in length and 0.5 µm in diameter, occurring as single rods or in short chains (Fig. S2). Colonies of *Ff. cerealis* C5^T^ grown on MRS agar were 1.5–2.0 mm in diameter, white, smooth, circular and convex. Cells were Gram-positive rods, ~1.5 µm long and 1.0 µm wide (Fig. S3).

**Fig. 2.**
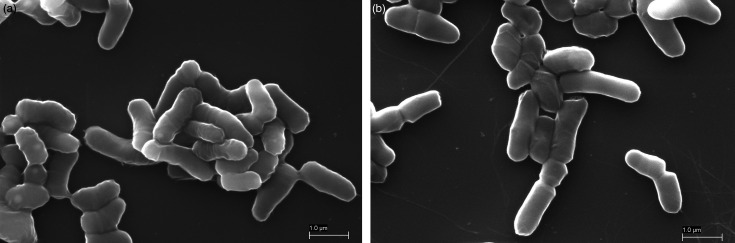
Scanning electron micrographs of (**a**) *Ff. cerevisiae* LTH 5742^T^ and (**b**) *Ff. cerealis* C5^T^. Micrographs were obtained using a ZEISS EVO 15 SEM operated in high-vacuum mode with secondary electron detection at 10 kV. Images were recorded at a magnification of ~30,000×.

In addition to light microscopy, scanning electron microscopy (SEM) was performed to visualize cell morphology and surface structures with high resolution. For sample preparation, colonies were suspended in PBS and pelleted by centrifugation at 13,000 ***g*** for 1 min at 4 °C. Pellets were washed with sterile demineralized water and fixed in 1% paraformaldehyde and 2.5% glutaraldehyde in 50 mM HEPES (pH 7.2) for 2 h at 4 °C. After fixation and repeated washing with water, a small volume of the cell suspension was applied to poly-l-lysine–coated glass slides, followed by dehydration through an ethanol series (30, 50, 70, 90 and 96%). Samples were sputter-coated with a 5 nm carbon layer and examined using a ZEISS EVO 15 scanning electron microscope (Carl Zeiss Microscopy GmbH) operated in high-vacuum mode. Secondary electrons were detected, at an accelerating voltage of 10 kV, to obtain high-resolution images of cell morphology. Representative SEM images of *Ff. cerevisiae* LTH 5742^T^ and *Ff. cerealis* C5^T^ are shown in Fig. 2(a, b), respectively.

In Fig. 2(a), SEM imaging of *Ff. cerevisiae* LTH 5742^T^ revealed rod-shaped cells with smooth surfaces and rounded ends. Cells measured ~1.2–2.0 µm in length and 0.4–0.6 µm in width. Surrounding many cells, a dense and irregular extracellular matrix was visible, consistent with the production of capsular EPSs reported for this strain [11]. This material frequently bridged adjacent cells. *Ff. cerealis* C5^T^ (Fig. 2b) appeared as slender, smooth-surfaced, rod-shaped cells with rounded ends, occurring both as single cells or in small, loosely associated groups. Cells measured ~1.5–2.3 µm in length and 0.4–0.6 µm in width. The overall morphology corresponded well with the dimensions and cell shape observed by light microscopy.

Growth of *Ff. cerevisiae* LTH 5742^T^ and *Ff. cerealis* C5^T^ at different temperatures was evaluated at 10, 15, 25, 30, 37 and 45 °C, as well as in MRS broth adjusted to pH values between 4.0 and 9.0 at 30 °C under anaerobic conditions (Figs S4 and S5). For all experiments, strains were inoculated into MRS broth at an initial optical density at 600 nm (OD₆₀₀) of 0.05 and incubated for 24 h; OD₆₀₀ values above 0.2 were considered indicative of growth to account for background variation. None of the two strains grew at 10 or 45 °C, while *Ff. cerevisiae* LTH 5742^T^ did not grow at 15 °C (Fig. S4). *Ff. cerevisiae* LTH 5742^T^ grew within a pH range of 4.0–7.0, but not at pH 8.0, while *Ff. cerealis* C5^T^ grew additionally at pH 8.0 and generally reached a higher OD₆₀₀_nm_ than LTH 5742^T^ (Fig. S5).

Information about carbohydrate utilization was obtained using an API 50 CH system (bioMérieux) for *Ff. cerealis* C5^T^ as previously described [11] together with data from previous publications (Table 6). All type strains can produce acid from d-glucose, d-mannose, *N*-acetylglucosamine, d-ribose and d-maltose and none of the strains fermented glycerol, erythritol, d-arabinose, l-xylose, d-adonitol, methyl *β*-d-xylopyranoside, l-sorbose, l-rhamnose, dulcitol, inositol, d-mannitol, d-sorbitol, methyl *α*-d-mannopyranoside, methyl *α*-d-glucopyranoside, amygdalin, arbutin, salicin, d-cellobiose, d-lactose, d-sucrose, d-trehalose, inulin, melezitose, d-raffinose, starch, glycogen, xylitol, gentiobiose, d-turanose, d-tagatose, d-fucose, l-fucose, l-arabitol, 2-ketogluconate or 5-ketogluconate. The strains LTH 5742^T^ and LTH 5750 exhibited identical carbohydrate utilization profiles and were able to ferment xylose, a trait shared with *Ff. rossiae* and *Ff. curtus*. The strain C5^T^ exhibited a distinct carbohydrate utilization pattern when compared to *Ff. milii*. It fermented melibiose but not d-lyxose, whereas *Ff. milii* is unable to utilize melibiose but can ferment lyxose.

**Table 6.** Carbohydrate utilization patterns of *Furfurilactobacillus* strains determined by API 50 CH after 48 h Sugars shown are ARA (l-arabinose), RIB (d-ribose), XYL (d-xylose), GAL (galactose), GLU (glucose), FRU (fructose), MNE (d-mannose), NAG (*N*-acetylglucosamine), MAL (maltose), MEL (melibiose), LYX (d-lyxose), GNT (potassium gluconate) and DARL (d-arabitol).

Strain	ARA	RIB	XYL	GLU	GAL	FRU	MNE	MEL	NAG	MAL	LYX	GNT	DARL
*Ff. rossiae* DSM 15814^T^	+	+	+	+	+	+	w	−	+	+	−	+	−
*Ff. milii* FUA3430^T^	+	+	−	+	+	+	+	−	+	+	+	+	−
*Ff. curtus* JCM 31185^T^	−	+	+	+	−	+	+	+	+	+	−	+	+
*Ff. siliginis* DSM 22696^T^	−	+	−	+	w	−	w	−	+	+	−	+	−
*‘Ff. entadae’* OKN 36^T^	w	+	−	+	w	+	w	−	+	+	+	+	−
*Ff. cerealis* C5^T*^	+	+	−	+	+	+	+	+	+	+	−	+	−
*Ff. cerevisiae* LTH 5742^T^	+	+	+	+	−	w	w	−	w	+	−	w	−
*Ff. cerevisiae* LTH 5750	+	+	+	+	−	w	w	−	w	+	−	w	−

+, Strong acid production; w, weak acid production; –, no acid production. Data for the following strains is taken from the given sources *Ff. rossiae* DSM 15814T [2], *Ff. milii* FUA3430T [5], *Ff. curtus* JCM 31185T [4], *Ff. siliginis* DSM 22696T [3], ‘*Ff. entadae’* OKN 36T [6], *Ff. cerevisiae* LTH 5742T and LTH 5750 [11].

*Data obtained in this study.

## Description of *Furfurilactobacillus cerevisiae* sp. nov.

*Furfurilactobacillus cerevisiae* sp. nov. (ce.re.vi′si.ae. L. gen. n. *cerevisiae*, of beer).

Colonies are 0.5–1.0 mm in diameter after 72 h of anaerobic growth on MRS agar at 30 °C. The colonies are white, smooth, circular and convex; when EPSs are produced, colonies appear shiny and sticky. Cells are non-spore-forming, Gram-stain positive rods, ~1.5 µm long and ~0.5 µm in diameter, occurring as single cells or in short chains. Growth is observed between 15 and 37 °C, but not at 10 °C or 45 °C, as well as in the pH range of 4–8. Acid is produced from glucose, fructose, mannose, arabinose, ribose, xylose, maltose, *N*-acetylglucosamine and gluconate. Comparative genomics indicates the presence of *β*-glucosidase genes and the absence of the propanediol utilization operon (*pduCDE*).

The type strain LTH 5742^T^ (=DSM 120514^T^=LMG 34151^T^) was isolated from spoiled beer in Germany. Its genome size is ~3.00 Mbp and the G+C content of the genome is 43.5 mol%. The GenBank accession numbers of the sequences of the 16S rRNA gene and the genome are PX421037 and GCA_048961765, respectively.

## Description of *Furfurilactobacillus cerealis* sp. nov.

*Furfurilactobacillus cerealis* sp. nov. (ce.re.a’lis. L. fem. adj. *cerealis*, related to cereals).

Colonies on MRS agar after 72 h of anaerobic incubation at 30 °C are white, smooth, circular and convex, with a diameter of 0.5–1.0 mm. Cells are Gram-stain-positive, non-spore-forming rods, ~1.5–2.0 µm long and 0.5–1.0 µm wide, occurring singly or in short chains. Growth is observed between 15 and 37 °C and at pH values between 4.0 and 9.0, while no growth occurs at 10 °C or 45 °C. Acid is produced from glucose, mannose, fructose, galactose, arabinose, ribose, maltose, melibiose, *N*-acetylglucosamine and gluconate. Comparative genomic analyses indicate the presence of genes for *β*-glucosidases, glutathione reductase and lactaldehyde dehydrogenase, and the absence of the *pduCDE* operon.

The type strain C5^T^ (=DSM 120515^T^=LMG 34152^T^) was isolated from sourdough in Italy. Its genome size is ~2.73 Mbp and the G+C content of the genome is 43.5 mol%. The GenBank accession numbers of the sequences of the 16S rRNA gene and the genome are PX394247 and GCF_009864005, respectively.

## Supplementary material

10.1099/ijsem.0.007009 Uncited Supplementary Material 1.

## Abbreviations

ANI: average nucleotide identity
dDDH: digital DNA–DNA hybridization
EPS: exopolysaccharide
iTOL: Interactive Tree of Life
MRS: de Man–Rogosa–Sharpe
SEM: scanning electron microscopy

## References

[1] Zheng J, Wittouck S, Salvetti E, Franz CMAP, Harris HMB (2020). A taxonomic note on the genus *Lactobacillus*: description of 23 novel genera, emended description of the genus *Lactobacillus* Beijerinck 1901, and union of *Lactobacillaceae* and *Leuconostocaceae*. Int J Syst Evol Microbiol.

[2] Corsetti A, Settanni L, van Sinderen D, Felis GE, Dellaglio F (2005). *Lactobacillus rossii* sp. nov., isolated from wheat sourdough. Int J Syst Evol Microbiol.

[3] Aslam Z, Im W-T, Ten LN, Lee M-J, Kim K-H (2006). Lactobacillus siliginis sp. nov., isolated from wheat sourdough in South Korea. Int J Syst Evol Microbiol.

[4] Asakawa Y, Takesue N, Asano S, Shimotsu S, Iijima K (2017). *Lactobacillus curtus* sp. nov., isolated from beer in Finland. Int J Syst Evol Microbiol.

[5] Simpson DJ, Zhang JS, D’Amico V, Llamas-Arriba MG, Gänzle MG (2022). *Furfurilactobacillus milii* sp. nov., isolated from fermented cereal foods. Int J Syst Evol Microbiol.

[6] Suzuki S, Okano K, Tamaki M, Tsujii Y, Endo A (2025). *Furfurilactobacillus entadae* sp. nov., Isolated from Bark of Entada phaseoloides. Curr Microbiol.

[7] Gaur G, Oh J-H, Filannino P, Gobbetti M, van Pijkeren J-P (2020). Genetic determinants of hydroxycinnamic acid metabolism in heterofermentative lactobacilli. Appl Environ Microbiol.

[8] De Angelis M, Bottacini F, Fosso B, Kelleher P, Calasso M (2014). *Lactobacillus rossiae*, a vitamin B12 producer, represents a metabolically versatile species within the genus *Lactobacillus*. PLoS One.

[9] Liang N, Zhao Z, Curtis JM, Gänzle MG (2022). Antifungal cultures and metabolites of lactic acid bacteria for use in dairy fermentations. Int J Food Microbiol.

[10] Schneiderbanger J, Jacob F, Hutzler M (2019). Genotypic and phenotypic diversity of *Lactobacillus rossiae* isolated from beer. J Appl Microbiol.

[11] Zipori D, Brychcy M, Weiss A, Schmidt H (2026). Selection and characterization of exopolysaccharide producing lactic acid bacteria as potential food starter cultures. Food Microbiol.

[12] Seemann T (2014). Prokka: rapid prokaryotic genome annotation. Bioinformatics.

[13] Page AJ, Cummins CA, Hunt M, Wong VK, Reuter S (2015). Roary: rapid large-scale prokaryote pan genome analysis. Bioinformatics.

[14] Price MN, Dehal PS, Arkin AP (2010). FastTree 2--approximately maximum-likelihood trees for large alignments. PLoS One.

[15] Letunic I, Bork P (2021). Interactive Tree Of Life (iTOL) v5: an online tool for phylogenetic tree display and annotation. Nucleic Acids Res.

[16] Lee I, Ouk Kim Y, Park S-C, Chun J (2016). OrthoANI: an improved algorithm and software for calculating average nucleotide identity. Int J Syst Evol Microbiol.

[17] Meier-Kolthoff JP, Carbasse JS, Peinado-Olarte RL, Göker M (2022). TYGS and LPSN: a database tandem for fast and reliable genome-based classification and nomenclature of prokaryotes. Nucleic Acids Res.

[18] Riesco R, Trujillo ME (2024). Update on the proposed minimal standards for the use of genome data for the taxonomy of prokaryotes. Int J Syst Evol Microbiol.

[19] Brynildsrud O, Bohlin J, Scheffer L, Eldholm V (2016). Erratum to: rapid scoring of genes in microbial pan-genome-wide association studies with Scoary. Genome Biol.

[20] Lin J, Xie J, Luo L, Gänzle M (2023). Characterization of GshAB of *Tetragenococcus halophilus*: a two-domain glutathione synthetase. Appl Microbiol Biotechnol.

[21] Gänzle MG (2015). Lactic metabolism revisited: metabolism of lactic acid bacteria in food fermentations and food spoilage. Cur Opin Food Sci.

[22] Jänsch A, Korakli M, Vogel RF, Gänzle MG (2007). Glutathione reductase from *Lactobacillus sanfranciscensis* DSM20451T: contribution to oxygen tolerance and thiol exchange reactions in wheat sourdoughs. Appl Environ Microbiol.

[23] Dymarska M, Widenmann A, Low KE, Abbott DW, Guan L (2024). Conversion of phytochemicals by lactobacilli: (Phospho)-β-glucosidases are specific for glucosylated phytochemicals rather than disaccharides. J Agric Food Chem.

[24] Altschul SF, Madden TL, Schäffer AA, Zhang J, Zhang Z (1997). Gapped BLAST and PSI-BLAST: a new generation of protein database search programs. Nucleic Acids Res.

[25] Pham VD, Gänzle MG (2025). *Fructilactobacillus frigidiflavus* sp. nov., a pigmented species, and *Levilactobacillus lettrarii* sp. nov., a propionate-producing species isolated from sourdough. Int J Syst Evol Microbiol.

[26] Liang N, Neužil-Bunešová V, Tejnecký V, Gänzle M, Schwab C (2021). 3-Hydroxypropionic acid contributes to the antibacterial activity of glycerol metabolism by the food microbe *Limosilactobacillus reuteri*. Food Microbiol.

[27] Dobson CM, Deneer H, Lee S, Hemmingsen S, Glaze S (2002). Phylogenetic analysis of the genus *Pediococcus*, including *Pediococcus claussenii* sp. nov., a novel lactic acid bacterium isolated from beer. Int J Syst Evol Microbiol.

[28] Sun Z, Harris HMB, McCann A, Guo C, Argimón S (2015). Expanding the biotechnology potential of lactobacilli through comparative genomics of 213 strains and associated genera. Nat Commun.

