# Supplementary material for: Proposal of Furfurilactobacillus cerevisiae sp. nov. isolated from spoiled beer and Furfurilactobacillus cerealis sp. nov. isolated from sourdough
Source: Int J Syst Evol Microbiol. 2025 Dec 18;75(12):007009. doi: 10.1099/ijsem.0.007009 (PMC12715276; doi:10.1099/ijsem.0.007009)
Supplement: Uncited Supplementary Material 1. [file ijsem-75-07009-s001.pdf]

Supplementary material

Proposal of *Furfurilactobacillus cerevisiae* sp. nov. isolated from spoiled beer and *Furfurilactobacillus cerealis* sp. nov. isolated from sourdough

Dor Zipori , Nanzhen Qiao, Merlin Brychcy, Michael Gänzle and Herbert Schmidt

Figure S1. Core-genome maximum-likelihood phylogenetic tree of *Furfurilactobacillus* genomes.

Figure S2. Gram-stained cells of *Ff. cerevisiae* LTH 5742^{T} under light microscope.

Figure S3. Gram-stained cells of *Ff. cerealis* $C5^{T}$ under light microscope.

Figure S4. Effect of temperature on the growth of *Ff. cerevisiae* LTH 5742^{T} and *Ff. cerealis* $C5^{T}$.

Figure S5. Effect of pH on the growth of *Ff. cerevisiae* LTH 5742^{T} and *Ff. cerealis* $C5^{T}$.

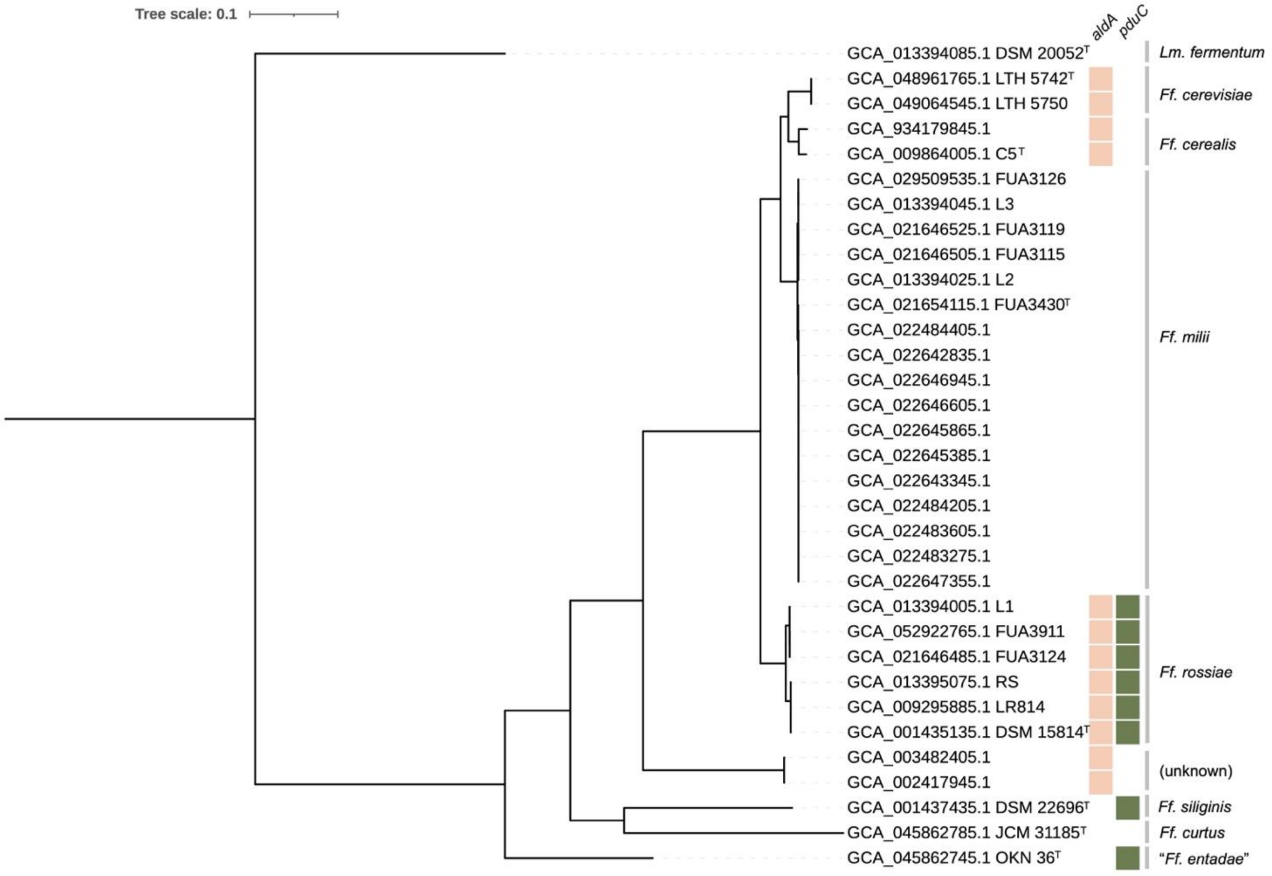

Figure S1. Core-genome maximum-likelihood phylogenetic tree of *Furfurilactobacillus* genomes.

All available *Furfurilactobacillus* genomes on NCBI (30 genomes in total after deduplication) and two additional genomes on GTDB (GCA_003482405.1 and GCA_002417945.1) that are not currently assigned to a named species are included, with *Lm. fermentum* DSM 20052^T rooted as the outgroup. A total of 182 core genes were identified with Roary using a cutoff of 60% identity. The phylogenetic tree was inferred using FastTree v2.1.11 under the JTT model. Bootstrap support for all nodes was greater than 99%. The heatmap depicts the binary presence and absence of genes *aldA* (KRM30456.1) and pduC (CAC82541.1) with thresholds of 40% identity and 70% coverage. The phylogenetic tree was visualized using iTOL (Letunic and Bork, 2021).

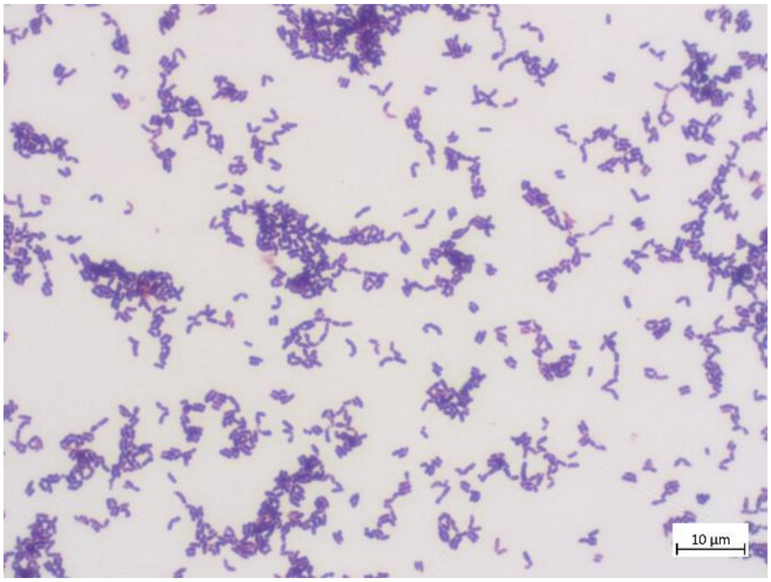

Figure S2. Gram-stained cells of *Ff. cerevisiae* LTH 5742^{T} under light microscope (Axio Lab.A1, Carl Zeiss) with 1000x magnification.

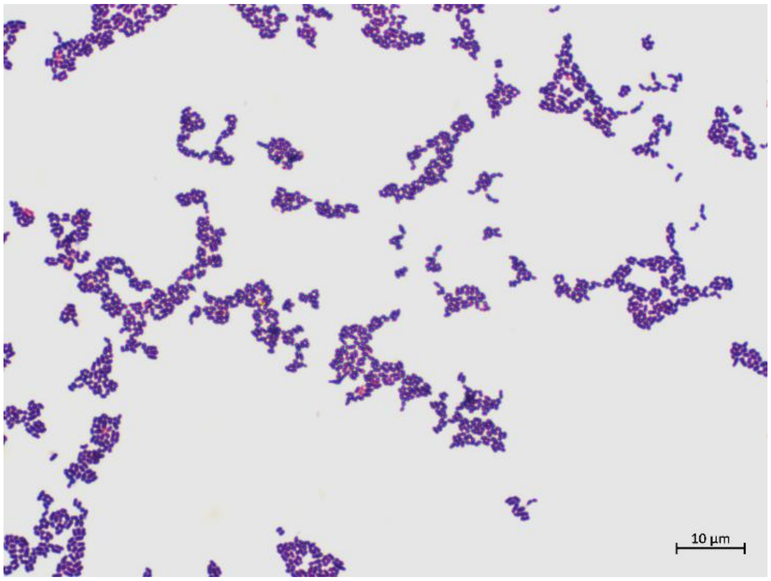

Figure S3. Gram-stained cells of *Ff. cerealis* C5^{T} under light microscope (Axio Lab.A1, Carl Zeiss) with 1000x magnification.

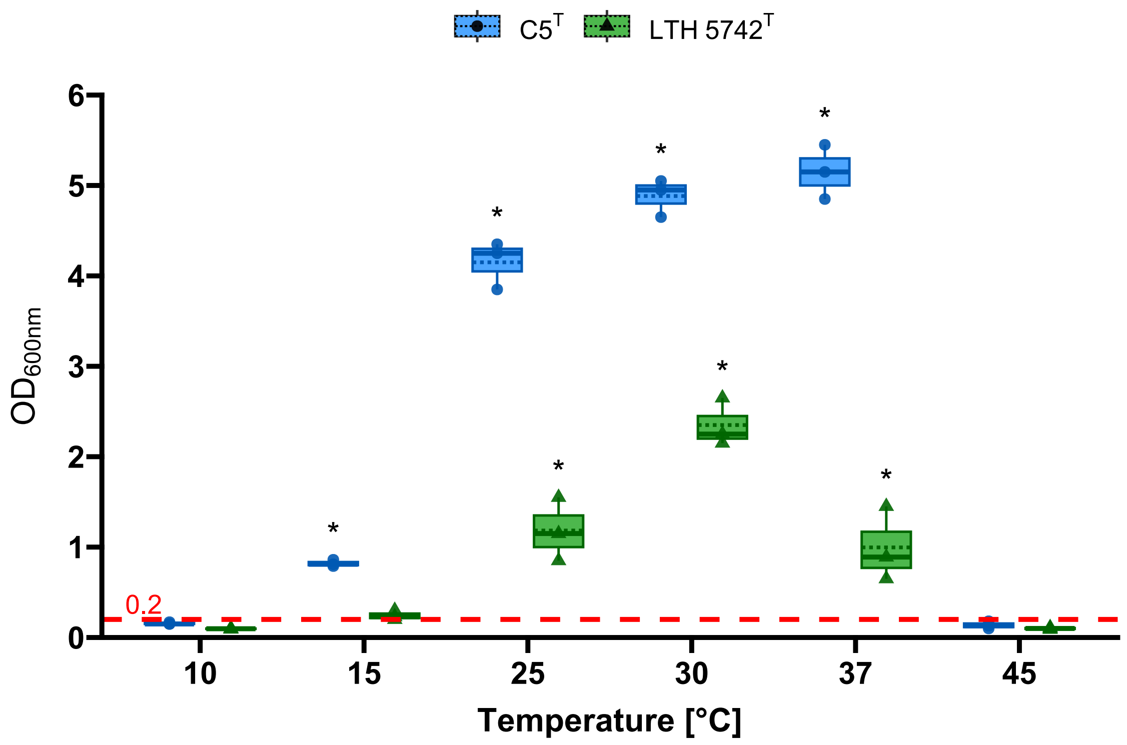

Figure S4. Effect of temperature on the growth of *Ff.* cerevisiae LTH 5742^T and *Ff. cerealis* C5^T

Optical density at a wavelength of 600 nm (OD_{600nm}) measured after 24 h of incubation in MRS broth under anaerobic conditions at the indicated temperatures. OD_{600} at t_0= 0.05. Boxplots represent the median, quartiles, and range; short-dashed lines inside boxes indicate the mean value. The red dashed horizontal line marks OD_{600} = 0.2, which was used as the threshold for significant growth. Asterisks (*) indicate $p < 0.05$ in a one-sample t-test against the 0.2 threshold. Each condition was tested in three independent biological replicates. Data was analyzed and plotted using R (v4.4.0) and RStudio (v2025.05.1) using tidyverse, ggprism.

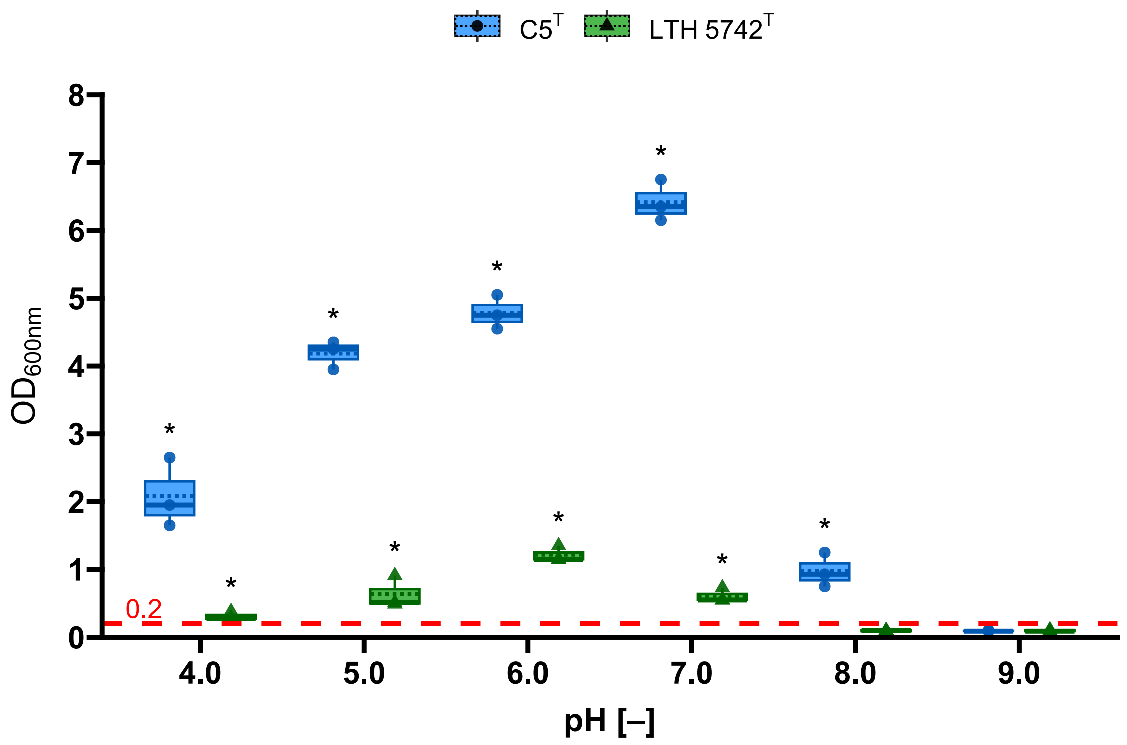

Figure S5. Effect of pH on the growth of on the growth of *Ff.* cerevisiae LTH 5742^T and *Ff. cerealis* C5^T

Optical density at a wavelength of 600 nm (OD_{600nm}) measured after 24 h of incubation in MRS broth at 30 °C under anaerobic conditions at the indicated pH values. The initial OD_{600} at t_0 was 0.05. Boxplots represent the median, quartiles, and range; short-dashed lines inside boxes indicate the mean value. The red dashed horizontal line marks OD_{600} = 0.2, which was used as the threshold for significant growth. Asterisks (*) indicate $p < 0.05$ in a one-sample t-test against the 0.2 threshold. Each condition was tested in three independent biological replicates. Data was analyzed and plotted using R (v4.4.0) and RStudio (v2025.05.1) using tidyverse, ggprism.
